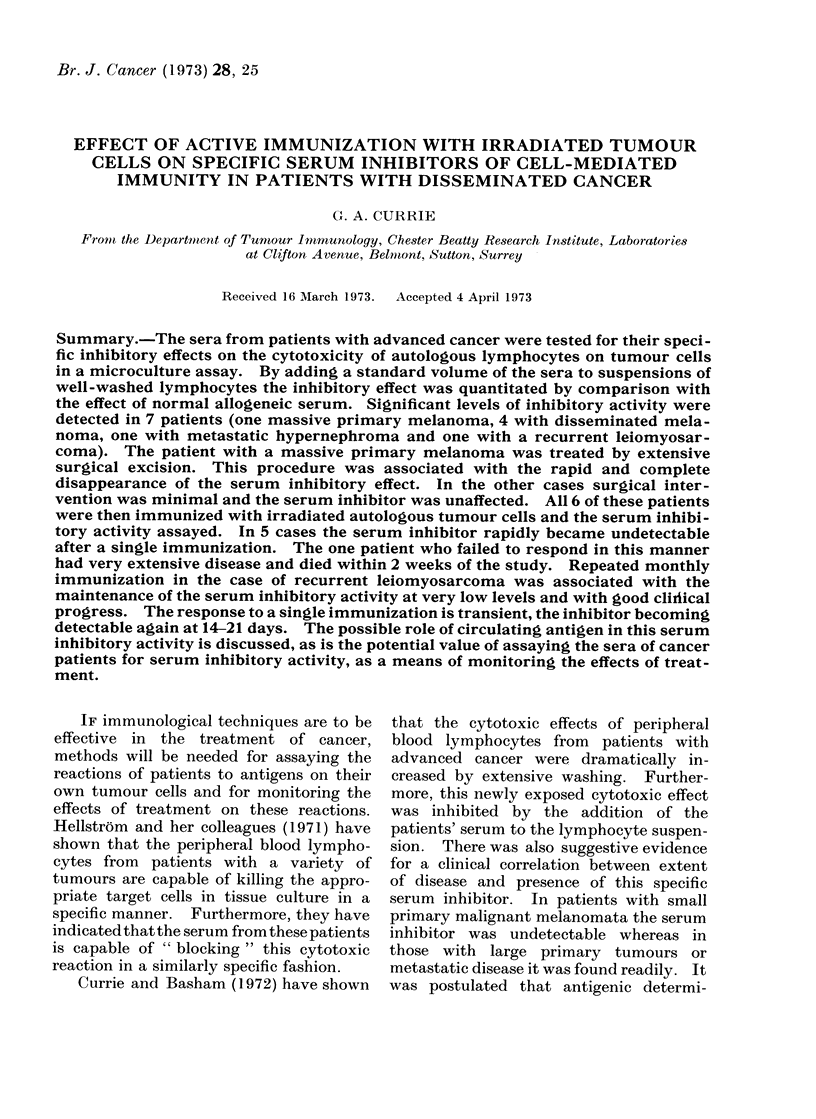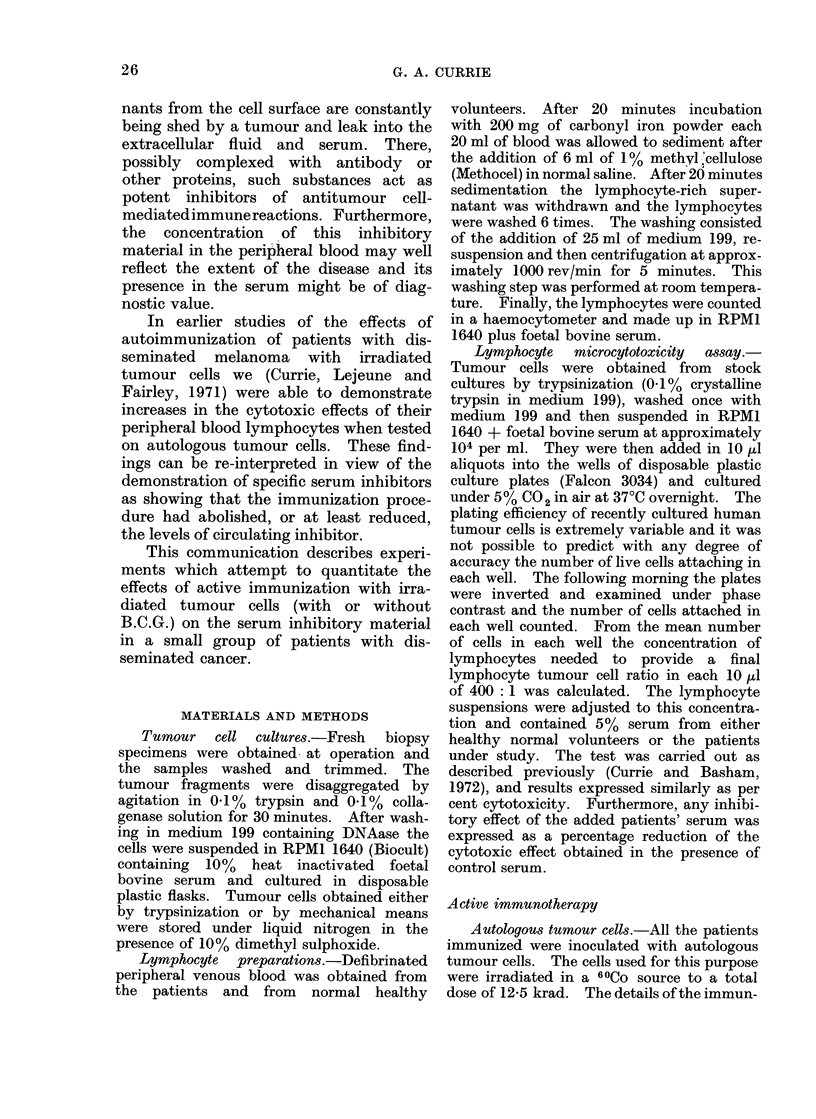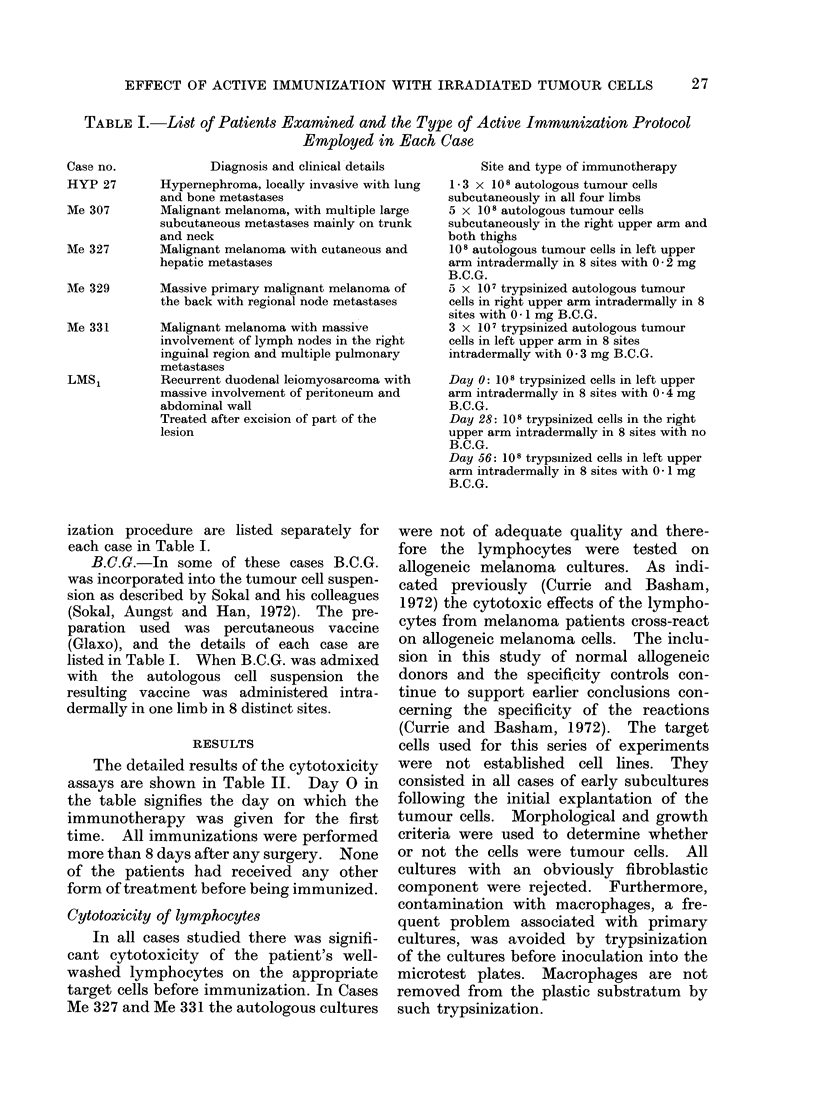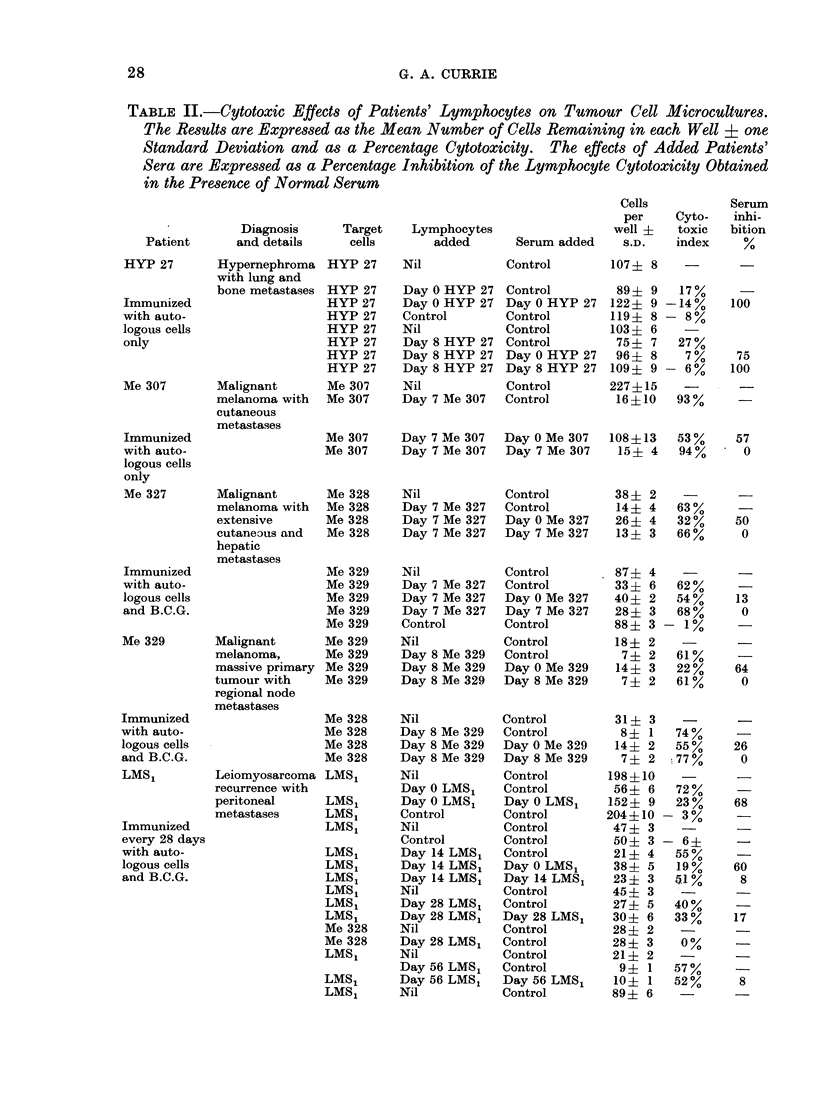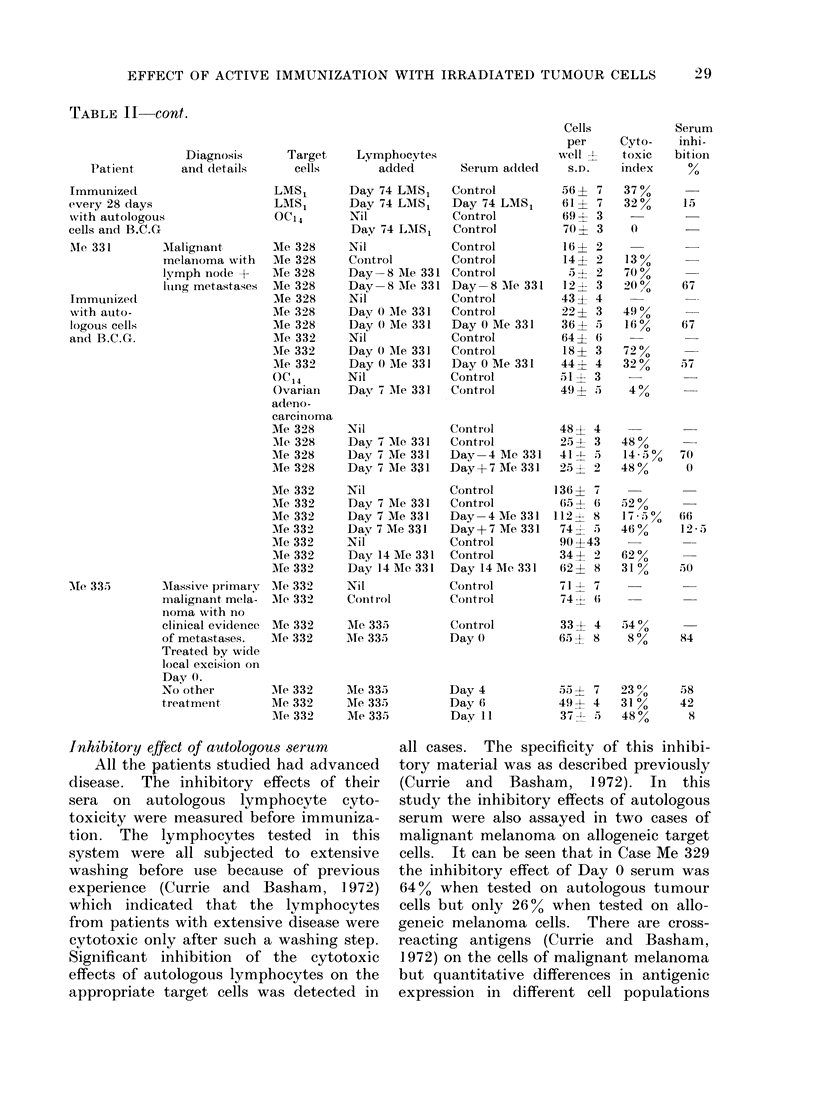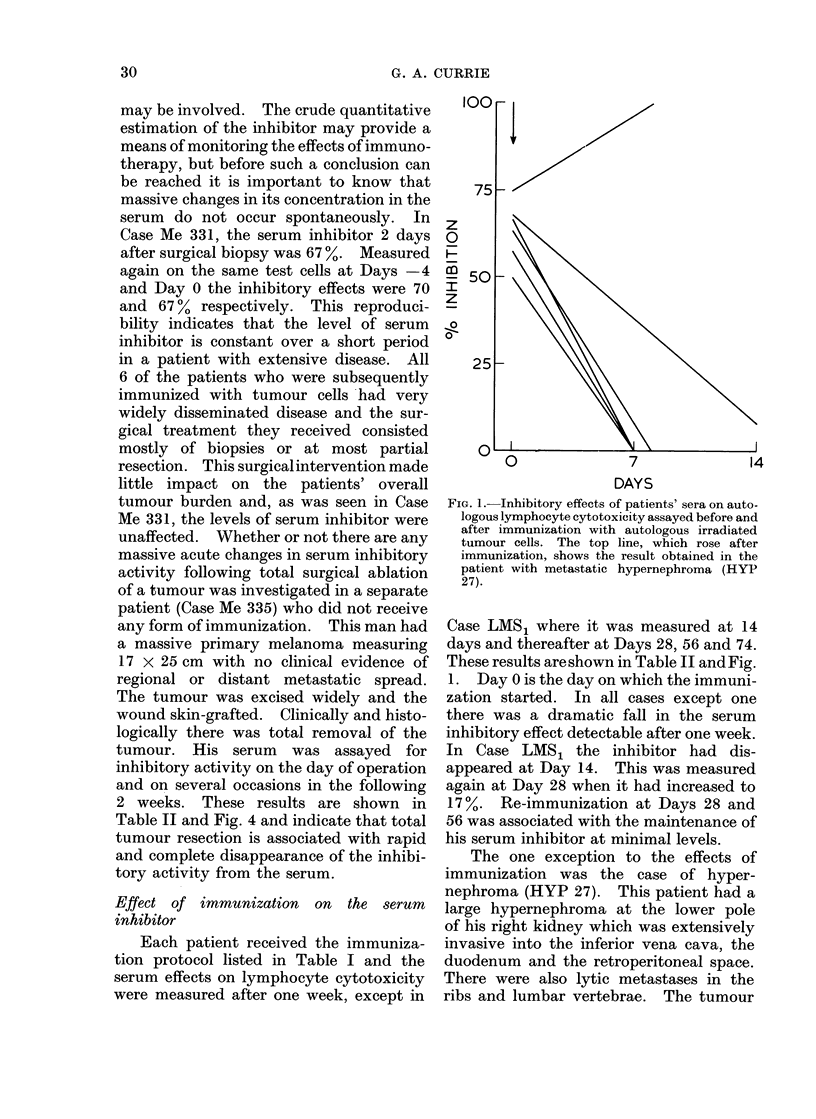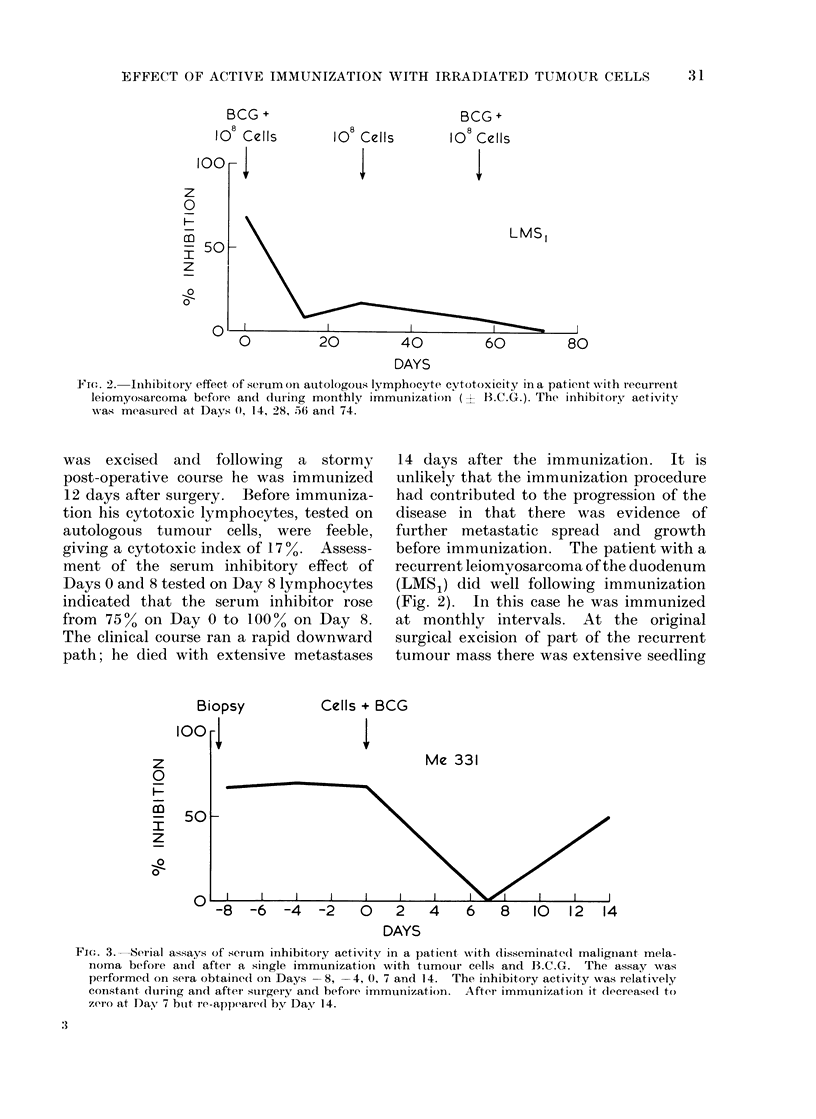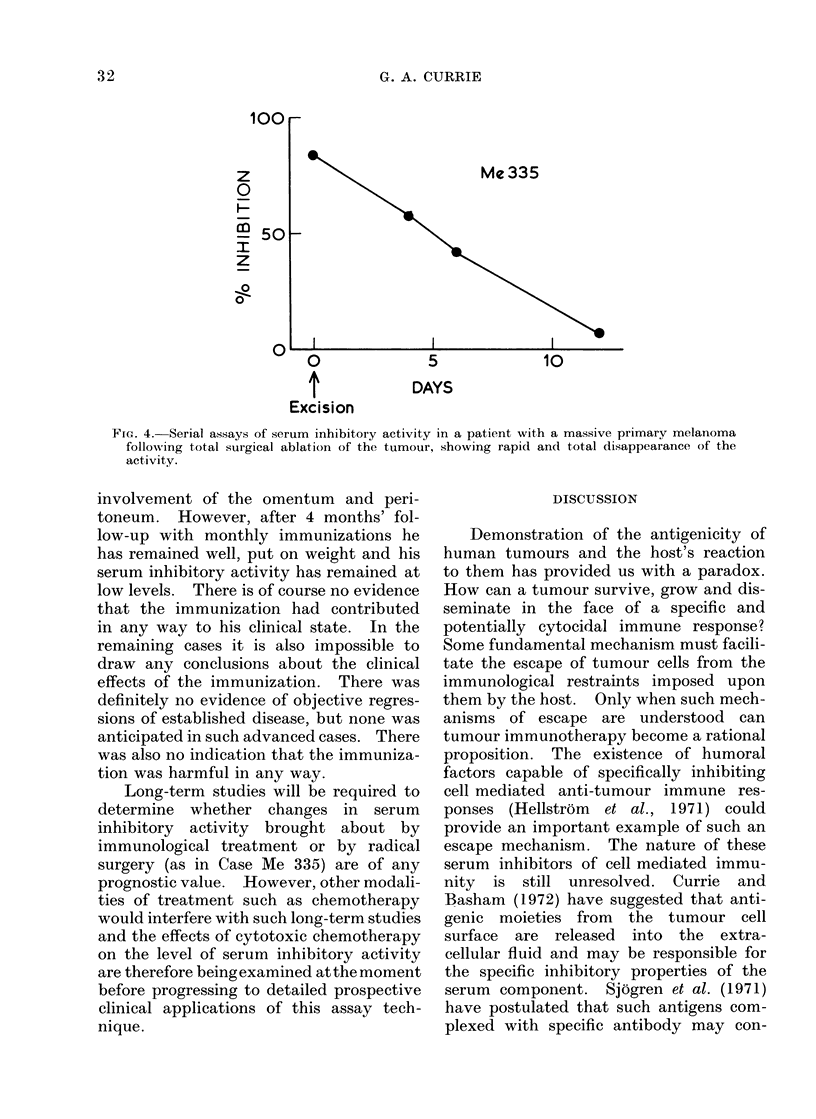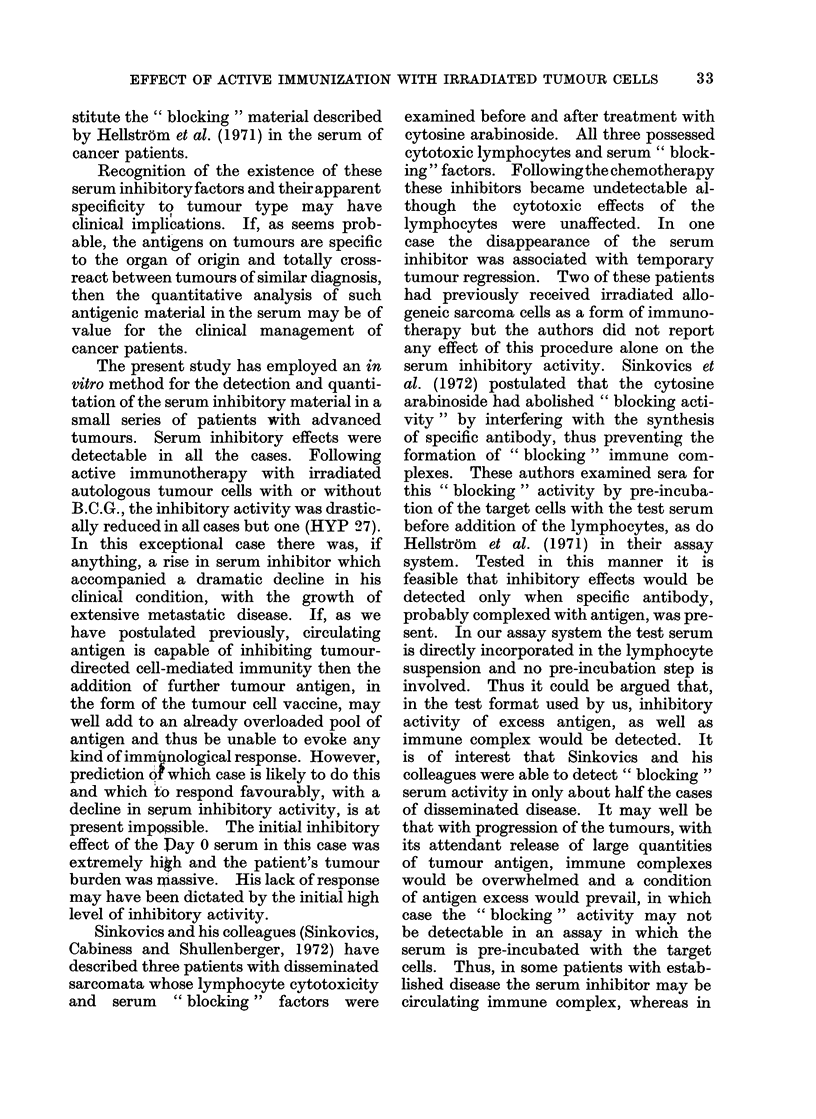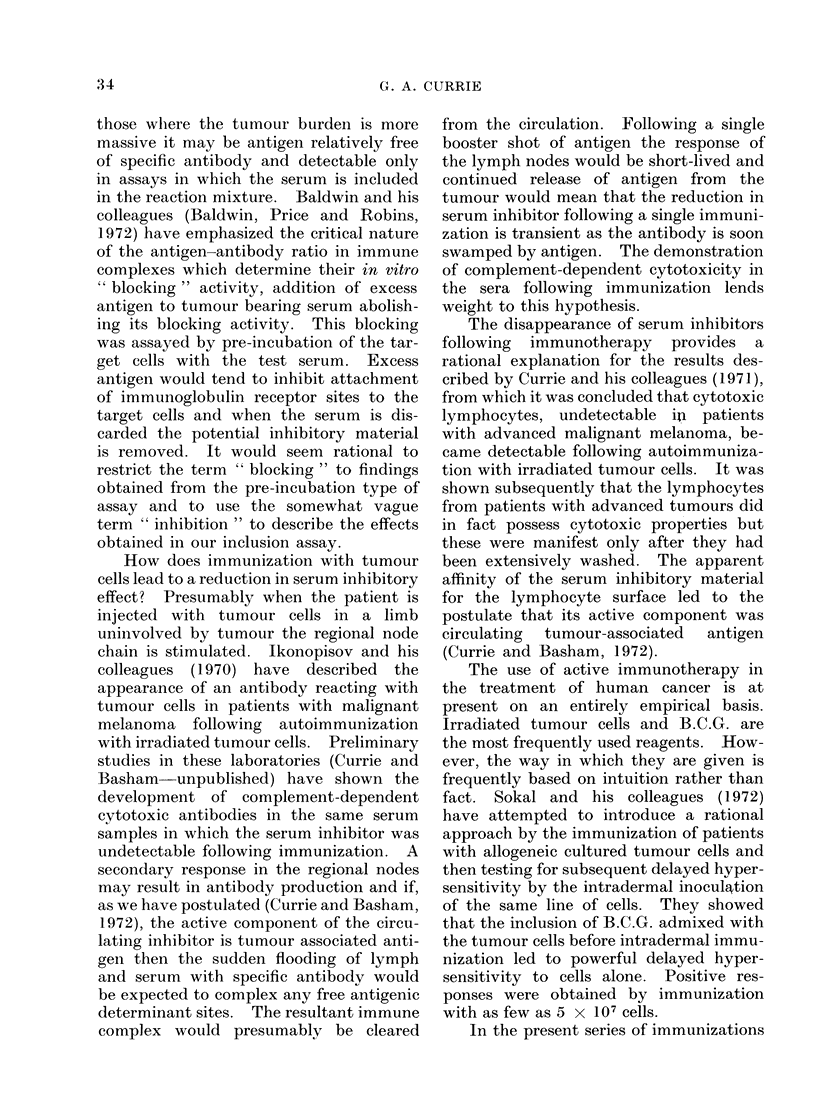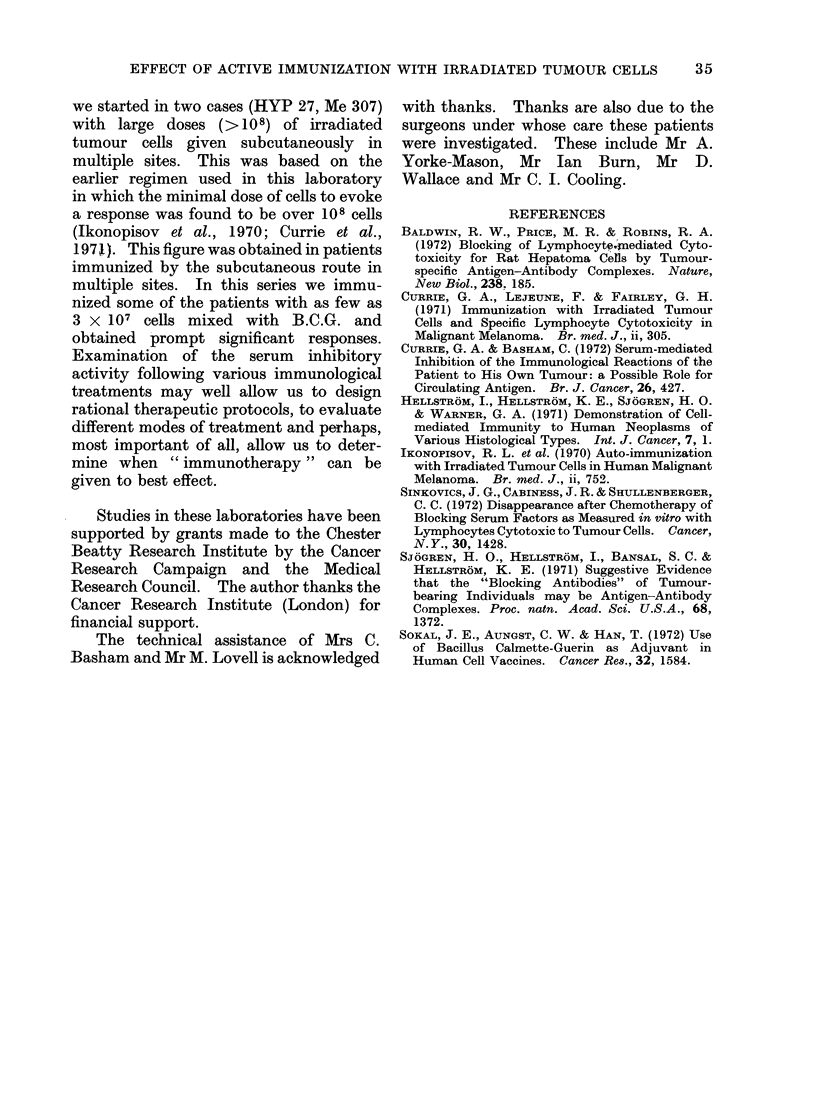# Effect of Active Immunization with Irradiated Tumour Cells on Specific Serum Inhibitors of Cell-mediated Immunity in Patients with Disseminated Cancer

**DOI:** 10.1038/bjc.1973.67

**Published:** 1973-07

**Authors:** G. A. Currie

## Abstract

The sera from patients with advanced cancer were tested for their specific inhibitory effects on the cytotoxicity of autologous lymphocytes on tumour cells in a microculture assay. By adding a standard volume of the sera to suspensions of well-washed lymphocytes the inhibitory effect was quantitated by comparison with the effect of normal allogeneic serum. Significant levels of inhibitory activity were detected in 7 patients (one massive primary melanoma, 4 with disseminated melanoma, one with metastatic hypernephroma and one with a recurrent leiomyosarcoma). The patient with a massive primary melanoma was treated by extensive surgical excision. This procedure was associated with the rapid and complete disappearance of the serum inhibitory effect. In the other cases surgical intervention was minimal and the serum inhibitor was unaffected. All 6 of these patients were then immunized with irradiated autologous tumour cells and the serum inhibitory activity assayed. In 5 cases the serum inhibitor rapidly became undetectable after a single immunization. The one patient who failed to respond in this manner had very extensive disease and died within 2 weeks of the study. Repeated monthly immunization in the case of recurrent leiomyosarcoma was associated with the maintenance of the serum inhibitory activity at very low levels and with good clinical progress. The response to a single immunization is transient, the inhibitor becoming detectable again at 14-21 days. The possible role of circulating antigen in this serum inhibitory activity is discussed, as is the potential value of assaying the sera of cancer patients for serum inhibitory activity, as a means of monitoring the effects of treatment.


Br. J. Cancer (1973) 28, 25

### EFFECT OF ACTIVE IMMUNIZATION WITH IRRADIATED TUMOUR CELLS ON SPECIFIC SERUM INHIBITORS OF CELL-MEDIATED IMMUNITY IN PATIENTS WITH DISSEMINATED CANCER

G. A. CURRIE

*From the Department of Tumour Immunology, Chester Beatty Research Institute, Laboratories at Clifton Avenue, Belmont, Sutton, Surrey*

Received 16 March 1973. Accepted 4 April 1973

**Summary.—The sera from patients with advanced cancer were tested for their specific inhibitory effects on the cytotoxicity of autologous lymphocytes on tumour cells in a microculture assay. By adding a standard volume of the sera to suspensions of well-washed lymphocytes the inhibitory effect was quantitated by comparison with the effect of normal allogeneic serum. Significant levels of inhibitory activity were detected in 7 patients (one massive primary melanoma, 4 with disseminated melanoma, one with metastatic hypernephroma and one with a recurrent leiomyosarcoma). The patient with a massive primary melanoma was treated by extensive surgical excision. This procedure was associated with the rapid and complete disappearance of the serum inhibitory effect. In the other cases surgical intervention was minimal and the serum inhibitor was unaffected. All 6 of these patients were then immunized with irradiated autologous tumour cells and the serum inhibitory activity assayed. In 5 cases the serum inhibitor rapidly became undetectable after a single immunization. The one patient who failed to respond in this manner had very extensive disease and died within 2 weeks of the study. Repeated monthly immunization in the case of recurrent leiomyosarcoma was associated with the maintenance of the serum inhibitory activity at very low levels and with good clinical progress. The response to a single immunization is transient, the inhibitor becoming detectable again at 14–21 days. The possible role of circulating antigen in this serum inhibitory activity is discussed, as is the potential value of assaying the sera of cancer patients for serum inhibitory activity, as a means of monitoring the effects of treatment.**

IF immunological techniques are to be effective in the treatment of cancer, methods will be needed for assaying the reactions of patients to antigens on their own tumour cells and for monitoring the effects of treatment on these reactions. Hellström and her colleagues (1971) have shown that the peripheral blood lymphocytes from patients with a variety of tumours are capable of killing the appropriate target cells in tissue culture in a specific manner. Furthermore, they have indicated that the serum from these patients is capable of " blocking " this cytotoxic reaction in a similarly specific fashion.

Currie and Basham (1972) have shown that the cytotoxic effects of peripheral blood lymphocytes from patients with advanced cancer were dramatically increased by extensive washing. Furthermore, this newly exposed cytotoxic effect was inhibited by the addition of the patients' serum to the lymphocyte suspension. There was also suggestive evidence for a clinical correlation between extent of disease and presence of this specific serum inhibitor. In patients with small primary malignant melanomata the serum inhibitor was undetectable whereas in those with large primary tumours or metastatic disease it was found readily. It was postulated that antigenic determinants from the cell surface are constantly being shed by a tumour and leak into the extracellular fluid and serum. There, possibly complexed with antibody or other proteins, such substances act as potent inhibitors of antitumour cell-mediated immune reactions. Furthermore, the concentration of this inhibitory material in the peripheral blood may well reflect the extent of the disease and its presence in the serum might be of diagnostic value.

In earlier studies of the effects of autoimmunization of patients with disseminated melanoma with irradiated tumour cells we (Currie, Lejeune and Fairley, 1971) were able to demonstrate increases in the cytotoxic effects of their peripheral blood lymphocytes when tested on autologous tumour cells. These findings can be re-interpreted in view of the demonstration of specific serum inhibitors as showing that the immunization procedure had abolished, or at least reduced, the levels of circulating inhibitor.

This communication describes experiments which attempt to quantitate the effects of active immunization with irradiated tumour cells (with or without B.C.G.) on the serum inhibitory material in a small group of patients with disseminated cancer.

### MATERIALS AND METHODS

*Tumour cell cultures*.—Fresh biopsy specimens were obtained at operation and the samples washed and trimmed. The tumour fragments were disaggregated by agitation in 0·1% trypsin and 0·1% collagenase solution for 30 minutes. After washing in medium 199 containing DNAase the cells were suspended in RPMI 1640 (Biocult) containing 10% heat inactivated foetal bovine serum and cultured in disposable plastic flasks. Tumour cells obtained either by trypsinization or by mechanical means were stored under liquid nitrogen in the presence of 10% dimethyl sulphoxide.

*Lymphocyte preparations*.—Defibrinated peripheral venous blood was obtained from the patients and from normal healthy volunteers. After 20 minutes incubation with 200 mg of carbonyl iron powder each 20 ml of blood was allowed to sediment after the addition of 6 ml of 1% methyl cellulose (Methocel) in normal saline. After 20 minutes sedimentation the lymphocyte-rich supernatant was withdrawn and the lymphocytes were washed 6 times. The washing consisted of the addition of 25 ml of medium 199, resuspension and then centrifugation at approximately 1000 rev/min for 5 minutes. This washing step was performed at room temperature. Finally, the lymphocytes were counted in a haemocytometer and made up in RPMI 1640 plus foetal bovine serum.

*Lymphocyte microcytotoxicity assay*.—Tumour cells were obtained from stock cultures by trypsinization (0·1% crystalline trypsin in medium 199), washed once with medium 199 and then suspended in RPMI 1640 + foetal bovine serum at approximately $10^4$ per ml. They were then added in 10 $\mu$l aliquots into the wells of disposable plastic culture plates (Falcon 3034) and cultured under 5% $CO_2$ in air at 37°C overnight. The plating efficiency of recently cultured human tumour cells is extremely variable and it was not possible to predict with any degree of accuracy the number of live cells attaching in each well. The following morning the plates were inverted and examined under phase contrast and the number of cells attached in each well counted. From the mean number of cells in each well the concentration of lymphocytes needed to provide a final lymphocyte tumour cell ratio in each 10 $\mu$l of 400 : 1 was calculated. The lymphocyte suspensions were adjusted to this concentration and contained 5% serum from either healthy normal volunteers or the patients under study. The test was carried out as described previously (Currie and Basham, 1972), and results expressed similarly as per cent cytotoxicity. Furthermore, any inhibitory effect of the added patients' serum was expressed as a percentage reduction of the cytotoxic effect obtained in the presence of control serum.

### *Active immunotherapy*

*Autologous tumour cells*.—All the patients immunized were inoculated with autologous tumour cells. The cells used for this purpose were irradiated in a $^{60}$Co source to a total dose of 12·5 krad. The details of the immun-

TABLE I.—*List of Patients Examined and the Type of Active Immunization Protocol Employed in Each Case*

| Case no. | Diagnosis and clinical details | Site and type of immunotherapy |
|---|---|---|
| HYP 27 | Hypernephroma, locally invasive with lung and bone metastases | $1 \cdot 3 \times 10^8$ autologous tumour cells subcutaneously in all four limbs |
| Me 307 | Malignant melanoma, with multiple large subcutaneous metastases mainly on trunk and neck | $5 \times 10^8$ autologous tumour cells subcutaneously in the right upper arm and both thighs |
| Me 327 | Malignant melanoma with cutaneous and hepatic metastases | $10^8$ autologous tumour cells in left upper arm intradermally in 8 sites with 0·2 mg B.C.G. |
| Me 329 | Massive primary malignant melanoma of the back with regional node metastases | $5 \times 10^7$ trypsinized autologous tumour cells in right upper arm intradermally in 8 sites with 0·1 mg B.C.G. |
| Me 331 | Malignant melanoma with massive involvement of lymph nodes in the right inguinal region and multiple pulmonary metastases | $3 \times 10^7$ trypsinized autologous tumour cells in left upper arm in 8 sites intradermally with 0·3 mg B.C.G. |
| $LMS_1$ | Recurrent duodenal leiomyosarcoma with massive involvement of peritoneum and abdominal wall<br>Treated after excision of part of the lesion | *Day 0*: $10^8$ trypsinized cells in left upper arm intradermally in 8 sites with 0·4 mg B.C.G.<br>*Day 28*: $10^8$ trypsinized cells in the right upper arm intradermally in 8 sites with no B.C.G.<br>*Day 56*: $10^8$ trypsinized cells in left upper arm intradermally in 8 sites with 0·1 mg B.C.G. |

ization procedure are listed separately for each case in Table I.

*B.C.G.*—In some of these cases B.C.G. was incorporated into the tumour cell suspension as described by Sokal and his colleagues (Sokal, Aungst and Han, 1972). The preparation used was percutaneous vaccine (Glaxo), and the details of each case are listed in Table I. When B.C.G. was admixed with the autologous cell suspension the resulting vaccine was administered intradermally in one limb in 8 distinct sites.

### RESULTS

The detailed results of the cytotoxicity assays are shown in Table II. Day O in the table signifies the day on which the immunotherapy was given for the first time. All immunizations were performed more than 8 days after any surgery. None of the patients had received any other form of treatment before being immunized.

### *Cytotoxicity of lymphocytes*

In all cases studied there was significant cytotoxicity of the patient's well-washed lymphocytes on the appropriate target cells before immunization. In Cases Me 327 and Me 331 the autologous cultures were not of adequate quality and therefore the lymphocytes were tested on allogeneic melanoma cultures. As indicated previously (Currie and Basham, 1972) the cytotoxic effects of the lymphocytes from melanoma patients cross-react on allogeneic melanoma cells. The inclusion in this study of normal allogeneic donors and the specificity controls continue to support earlier conclusions concerning the specificity of the reactions (Currie and Basham, 1972). The target cells used for this series of experiments were not established cell lines. They consisted in all cases of early subcultures following the initial explantation of the tumour cells. Morphological and growth criteria were used to determine whether or not the cells were tumour cells. All cultures with an obviously fibroblastic component were rejected. Furthermore, contamination with macrophages, a frequent problem associated with primary cultures, was avoided by trypsinization of the cultures before inoculation into the microtest plates. Macrophages are not removed from the plastic substratum by such trypsinization.

TABLE II.—*Cytotoxic Effects of Patients' Lymphocytes on Tumour Cell Microcultures. The Results are Expressed as the Mean Number of Cells Remaining in each Well ± one Standard Deviation and as a Percentage Cytotoxicity. The effects of Added Patients' Sera are Expressed as a Percentage Inhibition of the Lymphocyte Cytotoxicity Obtained in the Presence of Normal Serum*

| Patient | Diagnosis and details | Target cells | Lymphocytes added | Serum added | Cells per well ± S.D. | Cyto-toxic index | Serum inhi-bition % |
|---|---|---|---|---|---|---|---|
| HYP 27 | Hypernephroma | HYP 27 | Nil | Control | 107 ± 8 | — | — |
| | with lung and | | | | | | |
| | bone metastases | HYP 27 | Day 0 HYP 27 | Control | 89 ± 9 | 17% | — |
| Immunized | | HYP 27 | Day 0 HYP 27 | Day 0 HYP 27 | 122 ± 9 | −14% | 100 |
| with auto- | | HYP 27 | Control | Control | 119 ± 8 | − 8% | |
| logous cells | | HYP 27 | Nil | Control | 103 ± 6 | — | |
| only | | HYP 27 | Day 8 HYP 27 | Control | 75 ± 7 | 27% | |
| | | HYP 27 | Day 8 HYP 27 | Day 0 HYP 27 | 96 ± 8 | 7% | 75 |
| | | HYP 27 | Day 8 HYP 27 | Day 8 HYP 27 | 109 ± 9 | − 6% | 100 |
| Me 307 | Malignant | Me 307 | Nil | Control | 227 ± 15 | — | — |
| | melanoma with | Me 307 | Day 7 Me 307 | Control | 16 ± 10 | 93% | — |
| | cutaneous | | | | | | |
| | metastases | | | | | | |
| Immunized | | Me 307 | Day 7 Me 307 | Day 0 Me 307 | 108 ± 13 | 53% | 57 |
| with auto- | | Me 307 | Day 7 Me 307 | Day 7 Me 307 | 15 ± 4 | 94% | 0 |
| logous cells | | | | | | | |
| only | | | | | | | |
| Me 327 | Malignant | Me 328 | Nil | Control | 38 ± 2 | — | — |
| | melanoma with | Me 328 | Day 7 Me 327 | Control | 14 ± 4 | 63% | — |
| | extensive | Me 328 | Day 7 Me 327 | Day 0 Me 327 | 26 ± 4 | 32% | 50 |
| | cutaneous and | Me 328 | Day 7 Me 327 | Day 7 Me 327 | 13 ± 3 | 66% | 0 |
| | hepatic | | | | | | |
| | metastases | | | | | | |
| Immunized | | Me 329 | Nil | Control | 87 ± 4 | — | — |
| with auto- | | Me 329 | Day 7 Me 327 | Control | 33 ± 6 | 62% | — |
| logous cells | | Me 329 | Day 7 Me 327 | Day 0 Me 327 | 40 ± 2 | 54% | 13 |
| and B.C.G. | | Me 329 | Day 7 Me 327 | Day 7 Me 327 | 28 ± 3 | 68% | 0 |
| | | Me 329 | Control | Control | 88 ± 3 | − 1% | — |
| Me 329 | Malignant | Me 329 | Nil | Control | 18 ± 2 | — | — |
| | melanoma, | Me 329 | Day 8 Me 329 | Control | 7 ± 2 | 61% | — |
| | massive primary | Me 329 | Day 8 Me 329 | Day 0 Me 329 | 14 ± 3 | 22% | 64 |
| | tumour with | Me 329 | Day 8 Me 329 | Day 8 Me 329 | 7 ± 2 | 61% | 0 |
| | regional node | | | | | | |
| | metastases | | | | | | |
| Immunized | | Me 328 | Nil | Control | 31 ± 3 | — | — |
| with auto- | | Me 328 | Day 8 Me 329 | Control | 8 ± 1 | 74% | — |
| logous cells | | Me 328 | Day 8 Me 329 | Day 0 Me 329 | 14 ± 2 | 55% | 26 |
| and B.C.G. | | Me 328 | Day 8 Me 329 | Day 8 Me 329 | 7 ± 2 | 77% | 0 |
| $LMS_1$ | Leiomyosarcoma | $LMS_1$ | Nil | Control | 198 ± 10 | — | — |
| | recurrence with | | Day 0 $LMS_1$ | Control | 56 ± 6 | 72% | — |
| | peritoneal | $LMS_1$ | Day 0 $LMS_1$ | Day 0 $LMS_1$ | 152 ± 9 | 23% | 68 |
| | metastases | $LMS_1$ | Control | Control | 204 ± 10 | − 3% | — |
| Immunized | | $LMS_1$ | Nil | Control | 47 ± 3 | — | — |
| every 28 days | | | Control | Control | 50 ± 3 | − 6 ± | — |
| with auto- | | $LMS_1$ | Day 14 $LMS_1$ | Control | 21 ± 4 | 55% | — |
| logous cells | | $LMS_1$ | Day 14 $LMS_1$ | Day 0 $LMS_1$ | 38 ± 5 | 19% | 60 |
| and B.C.G. | | $LMS_1$ | Day 14 $LMS_1$ | Day 14 $LMS_1$ | 23 ± 3 | 51% | 8 |
| | | $LMS_1$ | Nil | Control | 45 ± 3 | — | — |
| | | $LMS_1$ | Day 28 $LMS_1$ | Control | 27 ± 5 | 40% | — |
| | | $LMS_1$ | Day 28 $LMS_1$ | Day 28 $LMS_1$ | 30 ± 6 | 33% | 17 |
| | | Me 328 | Nil | Control | 28 ± 2 | — | — |
| | | Me 328 | Day 28 $LMS_1$ | Control | 28 ± 3 | 0% | — |
| | | $LMS_1$ | Nil | Control | 21 ± 2 | — | — |
| | | | Day 56 $LMS_1$ | Control | 9 ± 1 | 57% | — |
| | | $LMS_1$ | Day 56 $LMS_1$ | Day 56 $LMS_1$ | 10 ± 1 | 52% | 8 |
| | | $LMS_1$ | Nil | Control | 89 ± 6 | — | — |

TABLE II—*cont.*

| Patient | Diagnosis and details | Target cells | Lymphocytes added | Serum added | Cells per well ± S.D. | Cyto-toxic index | Serum inhi-bition % |
|---|---|---|---|---|---|---|---|
| Immunized | | $LMS_1$ | Day 74 $LMS_1$ | Control | 56 ± 7 | 37% | — |
| every 28 days | | $LMS_1$ | Day 74 $LMS_1$ | Day 74 $LMS_1$ | 61 ± 7 | 32% | 15 |
| with autologous | | $OC_{14}$ | Nil | Control | 69 ± 3 | — | — |
| cells and B.C.G | | | Day 74 $LMS_1$ | Control | 70 ± 3 | 0 | — |
| Me 331 | Malignant | Me 328 | Nil | Control | 16 ± 2 | — | — |
| | melanoma with | Me 328 | Control | Control | 14 ± 2 | 13% | — |
| | lymph node + | Me 328 | Day − 8 Me 331 | Control | 5 ± 2 | 70% | — |
| | lung metastases | Me 328 | Day − 8 Me 331 | Day − 8 Me 331 | 12 ± 3 | 20% | 67 |
| Immunized | | Me 328 | Nil | Control | 43 ± 4 | — | — |
| with auto- | | Me 328 | Day 0 Me 331 | Control | 22 ± 3 | 49% | — |
| logous cells | | Me 328 | Day 0 Me 331 | Day 0 Me 331 | 36 ± 5 | 16% | 67 |
| and B.C.G. | | Me 332 | Nil | Control | 64 ± 6 | — | — |
| | | Me 332 | Day 0 Me 331 | Control | 18 ± 3 | 72% | — |
| | | Me 332 | Day 0 Me 331 | Day 0 Me 331 | 44 ± 4 | 32% | 57 |
| | | $OC_{14}$ | Nil | Control | 51 ± 3 | — | — |
| | | Ovarian adeno-carcinoma | Day 7 Me 331 | Control | 49 ± 5 | 4% | — |
| | | Me 328 | Nil | Control | 48 ± 4 | — | — |
| | | Me 328 | Day 7 Me 331 | Control | 25 ± 3 | 48% | — |
| | | Me 328 | Day 7 Me 331 | Day − 4 Me 331 | 41 ± 5 | 14·5% | 70 |
| | | Me 328 | Day 7 Me 331 | Day + 7 Me 331 | 25 ± 2 | 48% | 0 |
| | | Me 332 | Nil | Control | 136 ± 7 | — | — |
| | | Me 332 | Day 7 Me 331 | Control | 65 ± 6 | 52% | — |
| | | Me 332 | Day 7 Me 331 | Day − 4 Me 331 | 112 ± 8 | 17·5% | 66 |
| | | Me 332 | Day 7 Me 331 | Day + 7 Me 331 | 74 ± 5 | 46% | 12·5 |
| | | Me 332 | Nil | Control | 90 ± 43 | — | — |
| | | Me 332 | Day 14 Me 331 | Control | 34 ± 2 | 62% | — |
| | | Me 332 | Day 14 Me 331 | Day 14 Me 331 | 62 ± 8 | 31% | 50 |
| Me 335 | Massive primary | Me 332 | Nil | Control | 71 ± 7 | — | — |
| | malignant mela-noma with no | Me 332 | Control | Control | 74 ± 6 | — | — |
| | clinical evidence | Me 332 | Me 335 | Control | 33 ± 4 | 54% | — |
| | of metastases. Treated by wide local excision on Day 0. | Me 332 | Me 335 | Day 0 | 65 ± 8 | 8% | 84 |
| | No other | Me 332 | Me 335 | Day 4 | 55 ± 7 | 23% | 58 |
| | treatment | Me 332 | Me 335 | Day 6 | 49 ± 4 | 31% | 42 |
| | | Me 332 | Me 335 | Day 11 | 37 ± 5 | 48% | 8 |

*Inhibitory effect of autologous serum*

All the patients studied had advanced disease. The inhibitory effects of their sera on autologous lymphocyte cytotoxicity were measured before immunization. The lymphocytes tested in this system were all subjected to extensive washing before use because of previous experience (Currie and Basham, 1972) which indicated that the lymphocytes from patients with extensive disease were cytotoxic only after such a washing step. Significant inhibition of the cytotoxic effects of autologous lymphocytes on the appropriate target cells was detected in all cases. The specificity of this inhibitory material was as described previously (Currie and Basham, 1972). In this study the inhibitory effects of autologous serum were also assayed in two cases of malignant melanoma on allogeneic target cells. It can be seen that in Case Me 329 the inhibitory effect of Day 0 serum was 64% when tested on autologous tumour cells but only 26% when tested on allogeneic melanoma cells. There are cross-reacting antigens (Currie and Basham, 1972) on the cells of malignant melanoma but quantitative differences in antigenic expression in different cell populations may be involved. The crude quantitative estimation of the inhibitor may provide a means of monitoring the effects of immunotherapy, but before such a conclusion can be reached it is important to know that massive changes in its concentration in the serum do not occur spontaneously. In Case Me 331, the serum inhibitor 2 days after surgical biopsy was 67%. Measured again on the same test cells at Days —4 and Day 0 the inhibitory effects were 70 and 67% respectively. This reproducibility indicates that the level of serum inhibitor is constant over a short period in a patient with extensive disease. All 6 of the patients who were subsequently immunized with tumour cells had very widely disseminated disease and the surgical treatment they received consisted mostly of biopsies or at most partial resection. This surgical intervention made little impact on the patients' overall tumour burden and, as was seen in Case Me 331, the levels of serum inhibitor were unaffected. Whether or not there are any massive acute changes in serum inhibitory activity following total surgical ablation of a tumour was investigated in a separate patient (Case Me 335) who did not receive any form of immunization. This man had a massive primary melanoma measuring 17 × 25 cm with no clinical evidence of regional or distant metastatic spread. The tumour was excised widely and the wound skin-grafted. Clinically and histologically there was total removal of the tumour. His serum was assayed for inhibitory activity on the day of operation and on several occasions in the following 2 weeks. These results are shown in Table II and Fig. 4 and indicate that total tumour resection is associated with rapid and complete disappearance of the inhibitory activity from the serum.

*Effect of immunization on the serum inhibitor*

Each patient received the immunization protocol listed in Table I and the serum effects on lymphocyte cytotoxicity were measured after one week, except in

FIG. 1.—Inhibitory effects of patients' sera on autologous lymphocyte cytotoxicity assayed before and after immunization with autologous irradiated tumour cells. The top line, which rose after immunization, shows the result obtained in the patient with metastatic hypernephroma (HYP 27).

Case $LMS_1$ where it was measured at 14 days and thereafter at Days 28, 56 and 74. These results are shown in Table II and Fig. 1. Day 0 is the day on which the immunization started. In all cases except one there was a dramatic fall in the serum inhibitory effect detectable after one week. In Case $LMS_1$ the inhibitor had disappeared at Day 14. This was measured again at Day 28 when it had increased to 17%. Re-immunization at Days 28 and 56 was associated with the maintenance of his serum inhibitor at minimal levels.

The one exception to the effects of immunization was the case of hypernephroma (HYP 27). This patient had a large hypernephroma at the lower pole of his right kidney which was extensively invasive into the inferior vena cava, the duodenum and the retroperitoneal space. There were also lytic metastases in the ribs and lumbar vertebrae. The tumour

FIG. 2.—Inhibitory effect of serum on autologous lymphocyte cytotoxicity in a patient with recurrent leiomyosarcoma before and during monthly immunization ($\pm$ B.C.G.). The inhibitory activity was measured at Days 0, 14, 28, 56 and 74.

was excised and following a stormy post-operative course he was immunized 12 days after surgery. Before immunization his cytotoxic lymphocytes, tested on autologous tumour cells, were feeble, giving a cytotoxic index of 17%. Assessment of the serum inhibitory effect of Days 0 and 8 tested on Day 8 lymphocytes indicated that the serum inhibitor rose from 75% on Day 0 to 100% on Day 8. The clinical course ran a rapid downward path; he died with extensive metastases 14 days after the immunization. It is unlikely that the immunization procedure had contributed to the progression of the disease in that there was evidence of further metastatic spread and growth before immunization. The patient with a recurrent leiomyosarcoma of the duodenum (LMS$_1$) did well following immunization (Fig. 2). In this case he was immunized at monthly intervals. At the original surgical excision of part of the recurrent tumour mass there was extensive seedling

FIG. 3.—Serial assays of serum inhibitory activity in a patient with disseminated malignant melanoma before and after a single immunization with tumour cells and B.C.G. The assay was performed on sera obtained on Days $-8$, $-4$, 0, 7 and 14. The inhibitory activity was relatively constant during and after surgery and before immunization. After immunization it decreased to zero at Day 7 but re-appeared by Day 14.

FIG. 4.—Serial assays of serum inhibitory activity in a patient with a massive primary melanoma following total surgical ablation of the tumour, showing rapid and total disappearance of the activity.

involvement of the omentum and peritoneum. However, after 4 months' follow-up with monthly immunizations he has remained well, put on weight and his serum inhibitory activity has remained at low levels. There is of course no evidence that the immunization had contributed in any way to his clinical state. In the remaining cases it is also impossible to draw any conclusions about the clinical effects of the immunization. There was definitely no evidence of objective regressions of established disease, but none was anticipated in such advanced cases. There was also no indication that the immunization was harmful in any way.

Long-term studies will be required to determine whether changes in serum inhibitory activity brought about by immunological treatment or by radical surgery (as in Case Me 335) are of any prognostic value. However, other modalities of treatment such as chemotherapy would interfere with such long-term studies and the effects of cytotoxic chemotherapy on the level of serum inhibitory activity are therefore being examined at the moment before progressing to detailed prospective clinical applications of this assay technique.

### DISCUSSION

Demonstration of the antigenicity of human tumours and the host's reaction to them has provided us with a paradox. How can a tumour survive, grow and disseminate in the face of a specific and potentially cytocidal immune response? Some fundamental mechanism must facilitate the escape of tumour cells from the immunological restraints imposed upon them by the host. Only when such mechanisms of escape are understood can tumour immunotherapy become a rational proposition. The existence of humoral factors capable of specifically inhibiting cell mediated anti-tumour immune responses (Hellström *et al.*, 1971) could provide an important example of such an escape mechanism. The nature of these serum inhibitors of cell mediated immunity is still unresolved. Currie and Basham (1972) have suggested that antigenic moieties from the tumour cell surface are released into the extracellular fluid and may be responsible for the specific inhibitory properties of the serum component. Sjögren *et al.* (1971) have postulated that such antigens complexed with specific antibody may constitute the " blocking " material described by Hellström *et al.* (1971) in the serum of cancer patients.

Recognition of the existence of these serum inhibitory factors and their apparent specificity to tumour type may have clinical implications. If, as seems probable, the antigens on tumours are specific to the organ of origin and totally cross-react between tumours of similar diagnosis, then the quantitative analysis of such antigenic material in the serum may be of value for the clinical management of cancer patients.

The present study has employed an *in vitro* method for the detection and quantitation of the serum inhibitory material in a small series of patients with advanced tumours. Serum inhibitory effects were detectable in all the cases. Following active immunotherapy with irradiated autologous tumour cells with or without B.C.G., the inhibitory activity was drastically reduced in all cases but one (HYP 27). In this exceptional case there was, if anything, a rise in serum inhibitor which accompanied a dramatic decline in his clinical condition, with the growth of extensive metastatic disease. If, as we have postulated previously, circulating antigen is capable of inhibiting tumour-directed cell-mediated immunity then the addition of further tumour antigen, in the form of the tumour cell vaccine, may well add to an already overloaded pool of antigen and thus be unable to evoke any kind of immunological response. However, prediction of which case is likely to do this and which to respond favourably, with a decline in serum inhibitory activity, is at present impossible. The initial inhibitory effect of the Day 0 serum in this case was extremely high and the patient's tumour burden was massive. His lack of response may have been dictated by the initial high level of inhibitory activity.

Sinkovics and his colleagues (Sinkovics, Cabiness and Shullenberger, 1972) have described three patients with disseminated sarcomata whose lymphocyte cytotoxicity and serum " blocking " factors were examined before and after treatment with cytosine arabinoside. All three possessed cytotoxic lymphocytes and serum " blocking" factors. Following the chemotherapy these inhibitors became undetectable although the cytotoxic effects of the lymphocytes were unaffected. In one case the disappearance of the serum inhibitor was associated with temporary tumour regression. Two of these patients had previously received irradiated allogeneic sarcoma cells as a form of immunotherapy but the authors did not report any effect of this procedure alone on the serum inhibitory activity. Sinkovics *et al.* (1972) postulated that the cytosine arabinoside had abolished " blocking activity " by interfering with the synthesis of specific antibody, thus preventing the formation of " blocking " immune complexes. These authors examined sera for this " blocking " activity by pre-incubation of the target cells with the test serum before addition of the lymphocytes, as do Hellström *et al.* (1971) in their assay system. Tested in this manner it is feasible that inhibitory effects would be detected only when specific antibody, probably complexed with antigen, was present. In our assay system the test serum is directly incorporated in the lymphocyte suspension and no pre-incubation step is involved. Thus it could be argued that, in the test format used by us, inhibitory activity of excess antigen, as well as immune complex would be detected. It is of interest that Sinkovics and his colleagues were able to detect " blocking " serum activity in only about half the cases of disseminated disease. It may well be that with progression of the tumours, with its attendant release of large quantities of tumour antigen, immune complexes would be overwhelmed and a condition of antigen excess would prevail, in which case the " blocking " activity may not be detectable in an assay in which the serum is pre-incubated with the target cells. Thus, in some patients with established disease the serum inhibitor may be circulating immune complex, whereas in those where the tumour burden is more massive it may be antigen relatively free of specific antibody and detectable only in assays in which the serum is included in the reaction mixture. Baldwin and his colleagues (Baldwin, Price and Robins, 1972) have emphasized the critical nature of the antigen–antibody ratio in immune complexes which determine their *in vitro* " blocking " activity, addition of excess antigen to tumour bearing serum abolishing its blocking activity. This blocking was assayed by pre-incubation of the target cells with the test serum. Excess antigen would tend to inhibit attachment of immunoglobulin receptor sites to the target cells and when the serum is discarded the potential inhibitory material is removed. It would seem rational to restrict the term " blocking " to findings obtained from the pre-incubation type of assay and to use the somewhat vague term " inhibition " to describe the effects obtained in our inclusion assay.

How does immunization with tumour cells lead to a reduction in serum inhibitory effect? Presumably when the patient is injected with tumour cells in a limb uninvolved by tumour the regional node chain is stimulated. Ikonopisov and his colleagues (1970) have described the appearance of an antibody reacting with tumour cells in patients with malignant melanoma following autoimmunization with irradiated tumour cells. Preliminary studies in these laboratories (Currie and Basham—unpublished) have shown the development of complement-dependent cytotoxic antibodies in the same serum samples in which the serum inhibitor was undetectable following immunization. A secondary response in the regional nodes may result in antibody production and if, as we have postulated (Currie and Basham, 1972), the active component of the circulating inhibitor is tumour associated antigen then the sudden flooding of lymph and serum with specific antibody would be expected to complex any free antigenic determinant sites. The resultant immune complex would presumably be cleared from the circulation. Following a single booster shot of antigen the response of the lymph nodes would be short-lived and continued release of antigen from the tumour would mean that the reduction in serum inhibitor following a single immunization is transient as the antibody is soon swamped by antigen. The demonstration of complement-dependent cytotoxicity in the sera following immunization lends weight to this hypothesis.

The disappearance of serum inhibitors following immunotherapy provides a rational explanation for the results described by Currie and his colleagues (1971), from which it was concluded that cytotoxic lymphocytes, undetectable in patients with advanced malignant melanoma, became detectable following autoimmunization with irradiated tumour cells. It was shown subsequently that the lymphocytes from patients with advanced tumours did in fact possess cytotoxic properties but these were manifest only after they had been extensively washed. The apparent affinity of the serum inhibitory material for the lymphocyte surface led to the postulate that its active component was circulating tumour-associated antigen (Currie and Basham, 1972).

The use of active immunotherapy in the treatment of human cancer is at present on an entirely empirical basis. Irradiated tumour cells and B.C.G. are the most frequently used reagents. However, the way in which they are given is frequently based on intuition rather than fact. Sokal and his colleagues (1972) have attempted to introduce a rational approach by the immunization of patients with allogeneic cultured tumour cells and then testing for subsequent delayed hypersensitivity by the intradermal inoculation of the same line of cells. They showed that the inclusion of B.C.G. admixed with the tumour cells before intradermal immunization led to powerful delayed hypersensitivity to cells alone. Positive responses were obtained by immunization with as few as $5 \times 10^7$ cells.

In the present series of immunizations we started in two cases (HYP 27, Me 307) with large doses ($>10^8$) of irradiated tumour cells given subcutaneously in multiple sites. This was based on the earlier regimen used in this laboratory in which the minimal dose of cells to evoke a response was found to be over $10^8$ cells (Ikonopisov *et al.*, 1970; Currie *et al.*, 1971). This figure was obtained in patients immunized by the subcutaneous route in multiple sites. In this series we immunized some of the patients with as few as $3 \times 10^7$ cells mixed with B.C.G. and obtained prompt significant responses. Examination of the serum inhibitory activity following various immunological treatments may well allow us to design rational therapeutic protocols, to evaluate different modes of treatment and perhaps, most important of all, allow us to determine when " immunotherapy " can be given to best effect.

Studies in these laboratories have been supported by grants made to the Chester Beatty Research Institute by the Cancer Research Campaign and the Medical Research Council. The author thanks the Cancer Research Institute (London) for financial support.

The technical assistance of Mrs C. Basham and Mr M. Lovell is acknowledged with thanks. Thanks are also due to the surgeons under whose care these patients were investigated. These include Mr A. Yorke-Mason, Mr Ian Burn, Mr D. Wallace and Mr C. I. Cooling.

### REFERENCES

BALDWIN, R. W., PRICE, M. R. & ROBINS, R. A. (1972) Blocking of Lymphocyte-mediated Cytotoxicity for Rat Hepatoma Cells by Tumour-specific Antigen–Antibody Complexes. *Nature, New Biol.*, **238**, 185.

CURRIE, G. A., LEJEUNE, F. & FAIRLEY, G. H. (1971) Immunization with Irradiated Tumour Cells and Specific Lymphocyte Cytotoxicity in Malignant Melanoma. *Br. med. J.*, ii, 305.

CURRIE, G. A. & BASHAM, C. (1972) Serum-mediated Inhibition of the Immunological Reactions of the Patient to His Own Tumour: a Possible Role for Circulating Antigen. *Br. J. Cancer*, **26**, 427.

HELLSTRÖM, I., HELLSTRÖM, K. E., SJÖGREN, H. O. & WARNER, G. A. (1971) Demonstration of Cell-mediated Immunity to Human Neoplasms of Various Histological Types. *Int. J. Cancer*, **7**, 1.

IKONOPISOV, R. L. *et al.* (1970) Auto-immunization with Irradiated Tumour Cells in Human Malignant Melanoma. *Br. med. J.*, ii, 752.

SINKOVICS, J. G., CABINESS, J. R. & SHULLENBERGER, C. C. (1972) Disappearance after Chemotherapy of Blocking Serum Factors as Measured *in vitro* with Lymphocytes Cytotoxic to Tumour Cells. *Cancer, N.Y.*, **30**, 1428.

SJÖGREN, H. O., HELLSTRÖM, I., BANSAL, S. C. & HELLSTRÖM, K. E. (1971) Suggestive Evidence that the "Blocking Antibodies" of Tumour-bearing Individuals may be Antigen–Antibody Complexes. *Proc. natn. Acad. Sci. U.S.A.*, **68**, 1372.

SOKAL, J. E., AUNGST, C. W. & HAN, T. (1972) Use of Bacillus Calmette-Guerin as Adjuvant in Human Cell Vaccines. *Cancer Res.*, **32**, 1584.